# Role of Mesenteric Defect Closure in Preventing Internal Hernias After Ileocecectomy With Kono-S Anastomosis

**DOI:** 10.7759/cureus.77585

**Published:** 2025-01-17

**Authors:** Mena Louis, Nathaniel Grabill, Firdous Khan, Joe Williams, Terence Jackson

**Affiliations:** 1 General Surgery, Northeast Georgia Medical Center Gainesville, Gainesville, USA; 2 Surgery, Northeast Georgia Medical Center Gainesville, Gainesville, USA; 3 School of Medicine, Medical College of Georgia, Augusta University, Augusta, USA; 4 Gastroenterology and Hepatology, Philadelphia College of Osteopathic Medicine, Suwanee, USA; 5 Hepatobiliary, Northeast Georgia Medical Center Gainesville, Gainesville, USA

**Keywords:** crohn’s disease, defect closure, gastrointestinal stricture, ileocecectomy, internal hernia, kono-s anastomosis, mesenteric defect, postoperative complications, small bowel obstruction

## Abstract

Crohn’s disease is a chronic inflammatory bowel condition that frequently leads to complications such as strictures and bowel obstruction, often necessitating surgical intervention. Surgical approaches like ileocecectomy and right colectomy are commonly performed, with an ongoing debate about whether to close the mesenteric defect during these procedures to prevent internal hernias and small bowel obstruction. In this case, a 39-year-old male individual with longstanding Crohn’s disease underwent robotic-assisted colon mobilization, ileocecectomy with Kono-S anastomosis, and gastrojejunostomy to address strictures. On postoperative day two, he developed nausea, vomiting, and abdominal distension. Imaging revealed a small bowel closed-loop obstruction, which prompted reoperation. Laparoscopy and exploratory laparotomy identified an internal hernia through the ileocolic mesocolon defect, which was repaired by closing the defect.

The closure of the mesenteric defect has been shown to significantly reduce the risk of internal hernias and small bowel obstruction. Numerous studies indicate that leaving the defect open can increase the likelihood of these complications, which often necessitate additional surgery. Concerns about tension and ischemia following defect closure have not been substantiated when appropriate techniques are used. Although literature specific to procedures like Kono-S anastomosis is limited, general surgical evidence supports mesenteric defect closure to mitigate the risk of postoperative complications.

Mesenteric defect closure during surgeries for Crohn’s disease reduces the risk of internal hernias and other postoperative complications without significantly increasing other surgical risks. Routine closure is therefore recommended to enhance patient outcomes and reduce the need for further interventions.

## Introduction

Crohn’s disease is a chronic inflammatory bowel condition characterized by a wide range of clinical manifestations, often requiring surgical intervention for disease-related complications [1]. Among the various complications, stricture formation and obstructive phenotypes are particularly challenging to manage [2]. These strictures can occur anywhere along the gastrointestinal tract but frequently involve the terminal ileum and ileocecal valve, leading to significant morbidity [3]. While medical therapy plays a crucial role in managing inflammation, surgery remains the definitive treatment when symptoms are refractory to initial medical management or when fibrotic strictures develop, causing bowel obstruction or other complications [4].

Ileocecectomy, a surgical procedure involving the resection of the ileocecal segment, is commonly performed in patients with Crohn’s disease who develop terminal ileal strictures [5]. Various anastomotic techniques have been developed to improve surgical outcomes and reduce disease recurrence at the anastomosis site [6]. One such technique is the Kono-S anastomosis, which has garnered attention for reducing postoperative recurrence at the anastomotic site [7]. This method involves creating a longitudinal incision along the antimesenteric border of the proximal and distal bowel segments, which are then sutured together to form a wide-lumen, tension-free anastomosis. By minimizing scar tissue formation and narrowing at the anastomotic site, the Kono-S technique addresses the challenges of stricturing phenotypes commonly seen in Crohn's disease, reducing the risk of recurrence and improving long-term outcomes [8].

The decision of whether to close the mesenteric defect following bowel resections, particularly in the context of Crohn’s disease surgery, has been a topic of ongoing debate [9]. Mesenteric defects, left open, pose the risk of internal herniation, a serious complication that can lead to small bowel obstruction and necessitate reoperation [10]. However, the potential risks associated with mesenteric defect closure, such as tension and ischemia, must also be considered [11]. The literature on this topic, while comprehensive in relation to more traditional anastomoses, is still evolving with respect to specialized techniques like the Kono-S anastomosis.

In this context, understanding the advantages and limitations of both the Kono-S anastomosis and mesenteric defect closure is essential for optimizing surgical outcomes in Crohn’s disease patients [12]. While the primary goal of surgery in Crohn’s disease is to alleviate symptoms and prevent recurrence, minimizing the risk of postoperative complications such as internal herniation remains an important aspect of postoperative management [13]. This article aims to explore the current evidence surrounding these surgical techniques and provide insight into best practices for managing Crohn’s disease-related strictures.

## Case presentation

A 39-year-old male individual with a long-standing history of Crohn’s disease presented with symptoms of gastric outlet obstruction and ileocolonic stricture. His disease was initially managed with medications such as Pentasa, Humira, and Stelara, but he experienced refractory responses to these treatments. He underwent multiple endoscopic balloon dilations for pyloric and duodenal stenosis, but the relief was temporary. Due to loss of insurance and subsequent inability to continue biologic therapy, his symptoms worsened, and he transitioned to a liquid diet, leading to significant weight loss and malnutrition.

The patient reported progressive abdominal pain, nausea, reflux, and recurrent episodes of vomiting, which initially involved brown-colored emesis and later bright red blood, prompting his presentation to the emergency department. His bowel movements remained stable at one to two times per day with small-volume rectal bleeding, which was a baseline for him.

**Figure 1:**
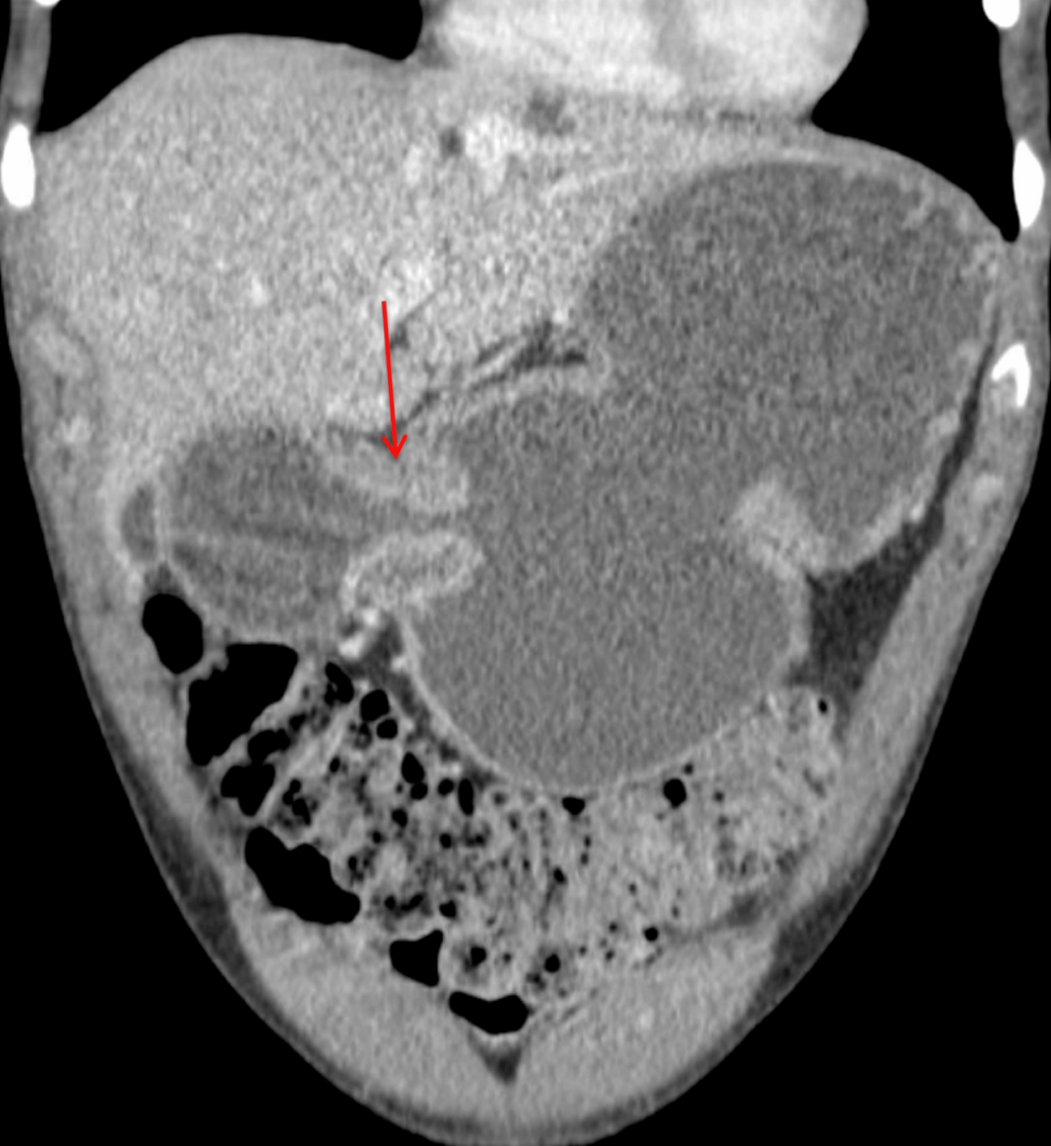
CT enterography (coronal view) with a distended stomach and stricture at the pyloric channel (red arrow) consistent with gastric outlet obstruction.

Physical examination revealed signs of malnutrition and intermittent abdominal distension. Diagnostic evaluations, including endoscopy and imaging, confirmed significant strictures at the ileocecal valve and pyloric channel, along with fibrotic narrowing and minimal active inflammation (Figure 1). Given his refractory disease and poor response to medical management, a surgical approach was deemed necessary.

The patient underwent a robotic-assisted colon mobilization, followed by an extracorporeal Kono-S anastomosis through a small midline incision to resect the affected ileocecal segment. A gastrojejunostomy was also performed to bypass the pyloric and duodenal strictures. The Kono-S anastomosis was chosen for its effectiveness in reducing recurrence at the anastomotic site. The surgery proceeded without initial complications, and the patient was closely monitored postoperatively.

The patient’s perioperative course included close monitoring and nutritional support to address pre-existing malnutrition and weight loss. Before surgery, total parenteral nutrition (TPN) was initiated to improve the patient’s nutritional status, given his inability to tolerate solid foods. Postoperatively, the patient remained on TPN while transitioning to a liquid diet as tolerated. Monitoring parameters included serial abdominal examinations, daily measurement of vital signs, and frequent laboratory assessments such as complete blood count (CBC), metabolic panel, and inflammatory markers to detect early signs of complications.

**Figure 2:**
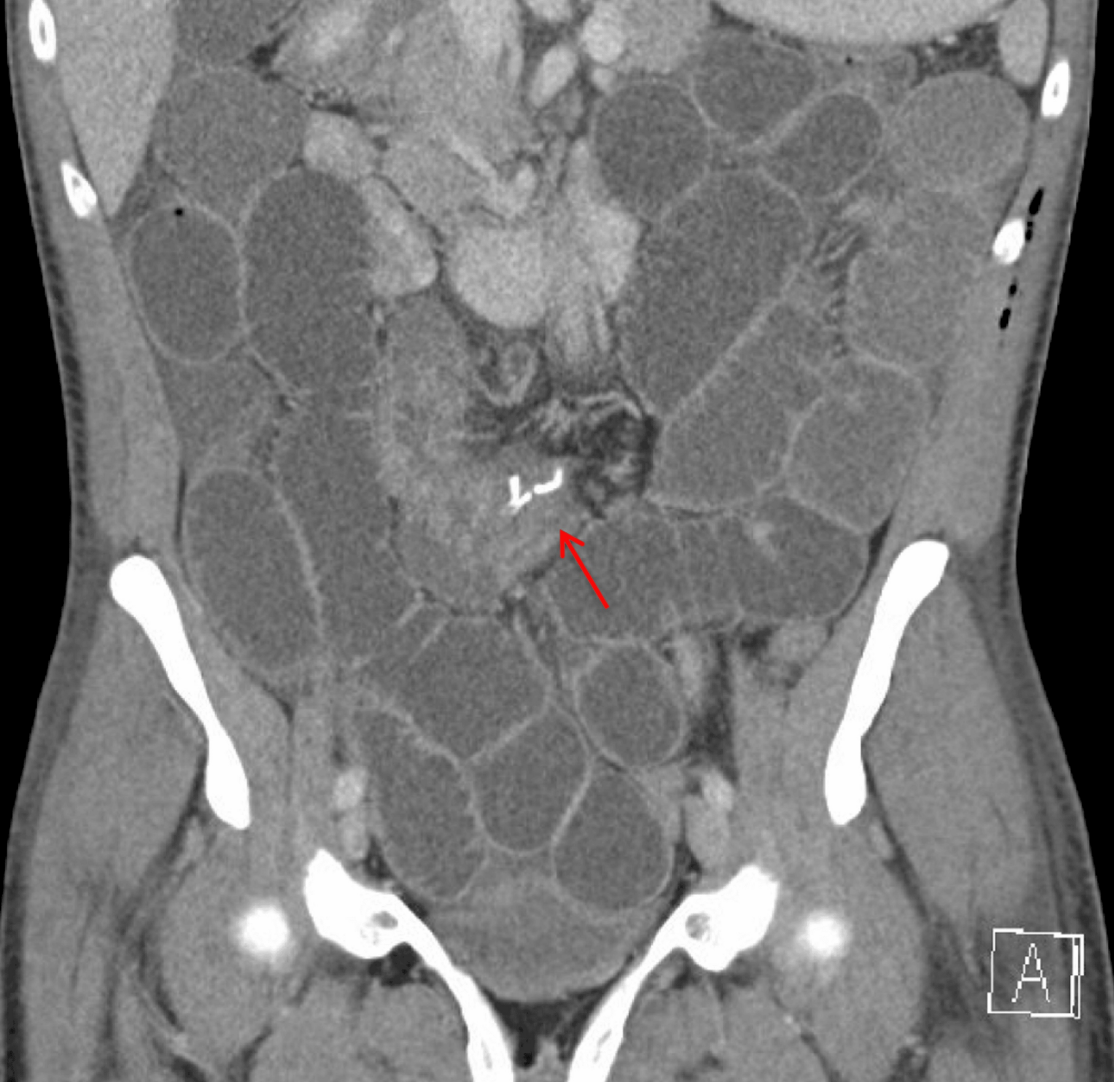
CT abdomen and pelvis with contrast (coronal view) shows diffuse dilated small bowel loops, a largely decompressed colon, and evidence of a transition zone near the anastomosis (red arrow). The transition zone represents the point where normal-caliber bowel transitions to dilated bowel, indicating the site of obstruction and serving as a critical finding in identifying the location of the underlying pathology.

**Figure 3:**
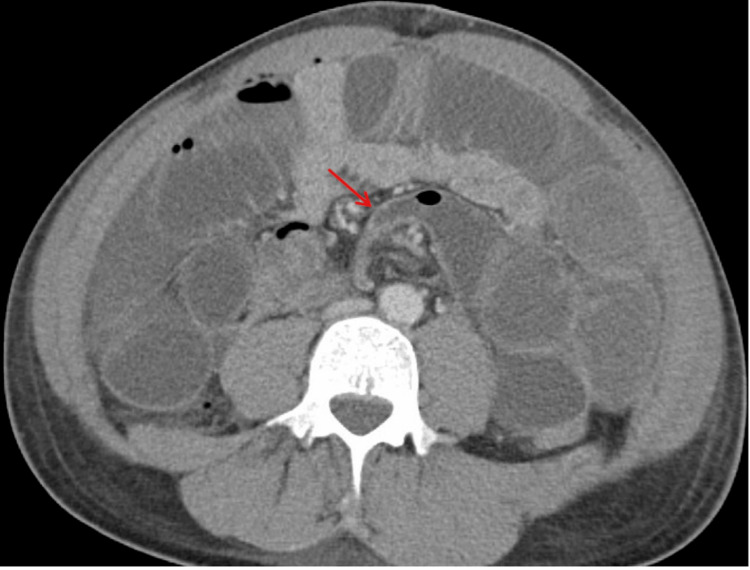
CT abdomen and pelvis with contrast (axial view) with evidence of a small bowel closed loop obstruction (red arrow) seen in the central portion of the abdomen in the mesentery, proximal to the surgical anastomosis.

On postoperative day 2, the patient developed nausea, vomiting, and abdominal distension. A CT scan was obtained, which showed evidence of a small bowel closed loop obstruction in the central portion of the abdomen, within the mesentery, proximal to the surgical anastomosis (Figures 2, 3). This raised concerns for an internal hernia or other postsurgical complications.

**Figure 4:**
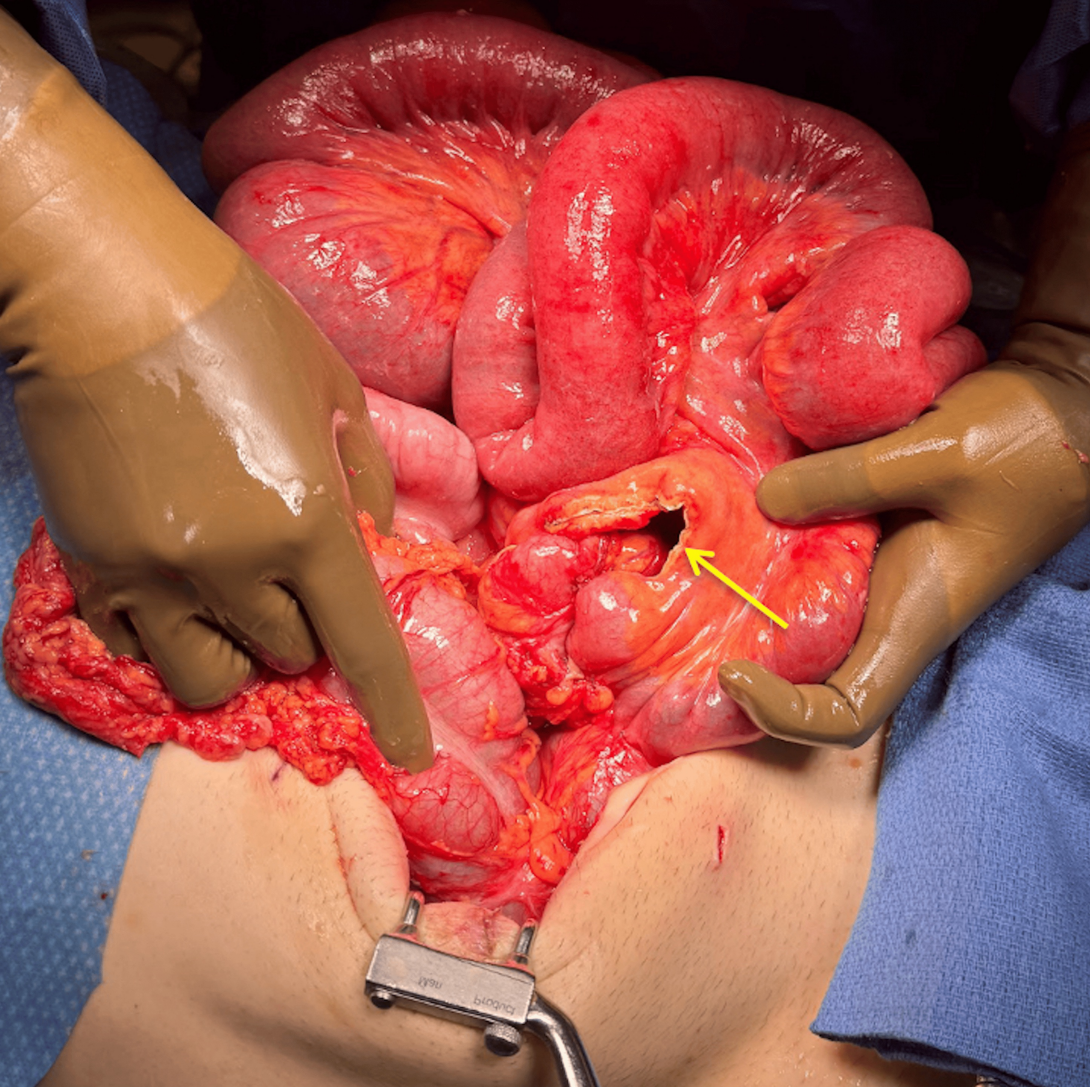
Intraoperative photo showing a mesenteric swirl (yellow arrow), a hallmark sign concerning for an internal hernia. This finding represents twisted mesenteric vessels, indicating bowel loops passing through a mesenteric defect, which was confirmed during surgery and subsequently repaired.

The patient was promptly taken back to the operating room for a diagnostic laparoscopy, followed by an exploratory laparotomy. The findings revealed an internal hernia of the right colon and terminal ileum through the ileocolic mesocolon defect (Figure 4). The herniated bowel was reduced, and the mesenteric defect was closed using interrupted silk sutures. There were no signs of ischemia, and the anastomoses at the ileocolonic and gastrojejunostomy sites were intact without any evidence of leakage. The patient tolerated diet advancement and was discharged on postoperative day 5.

## Discussion

Mesenteric defect closure has become an important consideration in gastrointestinal surgeries, particularly after ileocecectomy or right colectomy, due to its potential impact on postoperative complications such as internal hernias [14,15]. Internal herniation can lead to small bowel obstruction, a complication that often necessitates emergent reoperation and poses significant morbidity for patients [10,11]. This issue is particularly relevant in Crohn’s disease-related surgeries, where recurrent complications are already a concern [1,11].

Studies consistently demonstrate that leaving the mesenteric defect open significantly increases the risk of internal herniation and subsequent small bowel obstruction. Reported rates of internal hernias in such cases range from 2% to 8%, with higher risks noted in minimally invasive procedures [16,17]. These findings show the importance of routine mesenteric defect closure, supported by evidence from systematic reviews and studies, such as those published in the *British Journal of Surgery* [9,18,19]. Closure techniques have been shown to reduce reoperation rates and complications like bowel strangulation without introducing significant risks of ischemia or tension when performed correctly [10,18,20].

In the context of the Kono-S anastomosis, which is specifically designed to minimize anastomotic recurrence in Crohn’s disease patients, mesenteric defect closure is particularly relevant [21,22]. The Kono-S technique involves a functional end-to-end antimesenteric anastomosis, which creates a wide-lumen, tension-free connection to reduce stricture recurrence [23-26]. Although mesenteric defect closure is not explicitly integrated into the original technique, the principles of preventing internal herniation apply universally, particularly in surgeries addressing stricturing phenotypes of Crohn’s disease [12,27].

Several surgical societies, including the American Society of Colon and Rectal Surgeons (ASCRS) and the European Society of Coloproctology (ESCP), recommend mesenteric defect closure to minimize postoperative complications [28,29]. While literature specifically addressing mesenteric defect closure in Kono-S anastomosis is limited, general evidence supports its routine implementation, particularly given the increased risk of internal hernias in complex Crohn’s disease surgeries. In this case, failure to close the mesenteric defect contributed to small bowel obstruction, highlighting the clinical importance of this intervention [29,30].

The available evidence strongly supports routine mesenteric defect closure to enhance patient outcomes and prevent complications such as internal hernias and small bowel obstruction. While further research is needed to refine specific guidelines for Kono-S anastomosis, adopting mesenteric defect closure as standard practice in these surgeries aligns with best practices to improve long-term surgical outcomes [14,18,20].

## Conclusions

This case demonstrates the critical importance of mesenteric defect closure in preventing postoperative complications, such as internal hernias, following ileocecectomy with Kono-S anastomosis. The patient’s small bowel obstruction, resulting from an unclosed mesenteric defect, emphasizes the need for comprehensive surgical planning that considers both disease-specific factors and general postoperative risks. Incorporating defect closure in procedures like the Kono-S anastomosis can reduce complications and improve overall outcomes.
